# Combined inhibition of ACK1 and AKT shows potential toward targeted therapy against *KRAS*-mutant non-small-cell lung cancer

**DOI:** 10.17305/bjbms.2020.4746

**Published:** 2021-04

**Authors:** Xiangjing Yu, Jie Liu, Huawei Qiu, Huiting Hao, Jinhong Zhu, Shiyun Peng

**Affiliations:** 1 Clinical Laboratory, Harbin Medical University Cancer Hospital, Harbin, China; 2 Precision Medicine Center, Harbin Medical University Cancer Hospital, Harbin, China

**Keywords:** KRAS mutation, NSCLC, drug combination, Chou-Talalay, synergistic effect

## Abstract

Non-small-cell lung cancer (NSCLC) with Kirsten RAt Sarcoma 2 viral oncogene homolog (*KRAS*) mutation has become a clinical challenge in cancer treatment as *KRAS*-mutant tumors are often resistant to conventional anti-tumor therapies. Activated CDC42-associated kinase 1 (ACK1), an activator of protein kinase B (AKT), is a promising target for *KRAS*-mutant tumor therapy, but the downstream ACK1 signaling remains poorly understood. The aim of this study was to evaluate the effectiveness of combined ACK1/AKT inhibition on the proliferation, migration, invasion, and apoptosis of *KRAS*-mutant NSCLC cell lines (NCI-H23, NCI-H358, and A549). The cells were treated with an inhibitor of either ACK1 (dasatinib or sunitinib) or AKT (MK-2206 or GDC-0068), and the optimal concentrations of the two yielding synergistic tumor-killing effects were determined by applying the Chou-Talalay equation for drug combinations. We showed that combined administration of ACK1 and AKT inhibitors at the optimal concentrations effectively suppressed NSCLC cell viability and promoted apoptosis while inducing cell cycle arrest at the G2 phase. Moreover, NSCLC cell migration and invasion were inhibited by combined ACK1/AKT inhibition. These phenomena were associated with the reduced phosphorylation levels of ACK1 and AKT (at Ser473 and Thr308), as well as alterations in caspase-dependent apoptotic signaling. Collectively, our results demonstrate the promising therapeutic potential of combined ACK1/AKT inhibition as a strategy against *KRAS*-mutant NSCLC. Our findings provide the basis for the clinical translation of biological targeted drugs (ACK1 and AKT inhibitors) and their rational combination in cancer treatment.

## INTRODUCTION

Lung cancer is the most prevalent cancer worldwide, with estimated 228,000 newly diagnosed cases and causing 135,000 cancer-related deaths in the US in 2018 [1]. Most cases of lung cancer are classified as non-small-cell lung cancer (NSCLC), which constitutes approximately 85% of all cases, while the remaining 15% is identified as small-cell lung cancer [2]. In the past decade, two important sources of mutations in lung cancer have been discovered, namely Kirsten RAt Sarcoma 2 viral oncogene homolog (*KRAS*) and epidermal growth factor receptor (*EGFR*) mutations [3,4]. These mutations are the most common ones in lung cancer, especially NSCLC. Tumors with *KRAS* mutations generally have poor prognosis as they appear to be resistant to most available systemic therapies, making *KRAS* a key target for cancer treatment [5-7]. There is currently no approved *KRAS*-specific inhibitor for clinical use. Nevertheless, studies have shown that the combination of conventional chemotherapy with inhibitors of MEK, B-cell lymphoma-extra large (BCL-XL), and phosphoinositide 3-kinase is a promising method for the prevention and treatment of *KRAS*-mutant lung cancer [8,9].

Activated CDC42-associated kinase 1 (ACK1) is a widely expressed non-receptor tyrosine kinase that integrates and delivers signals from multiple tyrosine kinases, such as EGFR and platelet-derived growth factor receptor (PDGF-R) [10]. It has been shown to activate protein kinase B (AKT) by phosphorylating it at Tyr176 [11] and was also reportedly overexpressed and amplified in the tumorigenesis of different tissues such as stomach [12], prostate [13], and lung tissues [14]. In addition, ACK1 promotes the degradation of the tumor suppressor WW domain-containing oxidoreductase gene (*WWOX*), thus showing oncogenic properties [15,16]. The current evidence suggests that ACK1 overexpression is related to various tumors, including NSCLC, and that inhibiting ACK1 suppresses tumor cell invasion and metastasis [17,18]. The findings implicate that ACK1 is a promising target for tumor therapy, but the downstream ACK1 signaling remains poorly understood. The identification of an effective inhibitor of ACK1 and an understanding of its mechanism of action will provide a strong basis for the development of targeted treatment against *KRAS*-mutant NSCLC based on ACK1 inhibition.

The purpose of this study was to investigate effective targeted therapies for *KRAS*-mutant NSCLC. We combined inhibitors of ACK1 and AKT to suppress the over-activation of downstream signaling caused by *KRAS* mutation, thereby inhibiting tumor progression. We hypothesized that this combination is effective in inhibiting *KRAS*-mutant NSCLC cell growth and aimed to explore the specific molecular signaling pathways involved therein.

## MATERIALS AND METHODS

### Cell culture and drug treatment

The *KRAS*-mutant NSCLC cell lines NCI-H23, NCI-H358, and A549 were acquired from the cell bank of the Chinese Academy of Sciences (Shanghai, China). NCI-H23 and NCI-H358 cells were cultured in RPMI-1640 medium (SH30809.01B, Hyclone, Carlsbad, CA, USA) containing 10% fetal bovine serum (FBS, 10270-106, Gibco, Waltham, MA, USA) and 1% penicillin-streptomycin, whereas A549 cells were cultured in F12K medium (21127-022, Gibco, Waltham, MA) containing 10% FBS and 1% penicillin-streptomycin. All cells were maintained at 37°C in an incubator containing 5% CO_2_. For individual drug treatment, each cell type was incubated with inhibitors of ACK1 (dasatinib, A3017, APExBio, Houston, TX, USA; sunitinib, B1045, APExBio) or AKT (MK-2206, HY-10358, MCE; GDC-0068, A3006, APExBio). For combined drug treatment, NCI-H23 and NCI-H358 cells were incubated with sunitinib and GDC-0068 (both at IC_25_) while A549 cells were incubated with dasatinib and MK-2206 (both at IC_25_).

### 3-(4,5-dimethylthiazol-2-yl)-2,5-diphenyltetrazolium bromide (MTT) assay

An MTT assay (M1025, Solarbio, Beijing, China) was performed to identify the IC_25_ and IC_50_ (quarter and half maximal inhibitory concentration, respectively) of each drug in each *KRAS*-mutant NSCLC cell line. Cells were seeded in 96-well plates at 5 × 10^3^ cells per well and allowed to grow overnight at 37°C in an atmosphere containing 5% CO_2_. Thereafter, the cells were subjected to individual or combined drug treatment at the specified drug concentrations. After 48 hours of culture with the drug(s), 20 mL of MTT reagent (5 mg/mL) was added to each well and the plate was further incubated for 4 hours. The liquid was removed from the wells and 150 mL of dimethyl sulfoxide was added to each well. After 10 minutes of gentle shaking, the absorbance of the wells was measured using a plate reader (AMR-100, Allsheng, Hangzhou, China) at 490 nm.

### Flow cytometry

Flow cytometry was performed to assess the percentage of apoptotic cells and the proportion of cells in each phase of the cell cycle. The Annexin V-fluorescein isothiocyanate (FITC)/propidium iodide (PI) apoptosis assay kit (556547, BD Biosciences, Franklin Lakes, NJ, USA) was applied for the examination of apoptosis. Treated cells were trypsinized, centrifuged at 400 × g for 5 minutes at 4°C and resuspended in phosphate-buffered saline (PBS) at 1 × 10^5^ cells/mL. This step was repeated twice, after which 200 μL of binding buffer was added to the cells. Annexin V-FITC and PI (10 μL of each) were added to the cells and gently mixed. After 30 minutes of incubation at 4°C in the absence of light, 300 μL of binding buffer was added and the cells were immediately subjected to flow cytometry using a Novocyte apparatus (ACEA Biosciences, Inc., San Diego, CA). To evaluate cell cycle progression, treated cells were trypsinized and centrifuged at 400 × g for 5 minutes. The supernatant was discarded and the cells were resuspended in 300 μL of PBS. Then, 700 μL of anhydrous ethanol was added and the cells were fixed for 24 hours at -20°C. The fixed cells were centrifuged at 700 × g for 5 minutes, the supernatant was removed, and the cells were washed twice with cold PBS. The cells were then resuspended in 100 μL of 1 mg/mL RNAse A and incubated at 37°C for 30 minutes. Then, 400 μL of 50 μg/mL PI was added to the cells. After 10 minutes of staining in the absence of light, DNA content in the cells was measured using flow cytometry to assess the proportion of cells in various phases of the cell cycle. All flow cytometry results were analyzed using NovoExpress software (ACEA Biosciences, San Diego, CA, USA).

### Transwell assay

Transwell assays were carried out to evaluate the migration and invasion of treated cells. For the invasion experiments, Transwell inserts (Corning Inc., Corning, NY, USA) were first placed into the wells of a 24-well plate and coated with 80 μL of Matrigel (354230, BD Biosciences) at 37°C for 30 minutes (this step was not performed for migration experiments). Cells were treated accordingly and cultured for 24 hours in serum-free medium. They were then seeded in 500 μL of the medium into the top chamber of the Transwell inserts at 1 × 10^5^ cells per mL. Meanwhile, 750 μL of medium containing 10% FBS was added to the bottom chamber. The cells were then incubated at 37°C for 48 hours and the medium was removed. Thereafter, the cells were fixed in 1 mL of 4% paraformaldehyde in each well for 10 minutes at room temperature. The fixative was removed, the cells were washed once with PBS, and 1 mL of 0.5% crystal violet solution (PAB180004, Bioswamp, Wuhan, China) was added to each well. The cells were stained for 30 minutes and washed 3 times with PBS. Cells that have migrated or invaded to the bottom Transwell chambers were counted using an optical microscope.

### Western blot

Cells were washed twice with cold PBS and lysed at 4°C using radioimmunoprecipitation assay buffer containing protease and phosphatase inhibitors. The lysates were heated for 10 minutes at 95°C and centrifuged at 12,000 × *g* for 10 ­minutes, after which protein content was quantified using a bicinchoninic acid assay kit. For western blot, 20 μg of protein sample was loaded for sodium dodecyl sulfate-polyacrylamide gel electrophoresis. The separated proteins were transferred to polyvinylidene difluoride membranes that were pre-soaked in acetone for 5 minutes and cold electrophoresis buffer for 2 minutes. The membranes were blocked in 5% skim milk overnight at 4°C, incubated with primary antibodies overnight at 4°C, and incubated in horseradish peroxidase-conjugated goat anti-rabbit IgG secondary antibodies (1:20000, SAB43714, Bioswamp) for 1 hour at room temperature. Between each incubation, the membranes were washed 3 times with PBS/Tween 20 for 5 minutes each. After secondary antibody incubation, the membranes were subjected to enhanced chemiluminescent detection (WBKLS0010, Millipore, Billerica, MA, USA) using a Tanon-5200 analyzer (Tanon, Shanghai, China). Protein bands were visualized using Tanon GIS software. The following primary antibodies were used for western blot: phosphorylated (p)-ACK1 (1:1000, PAB40590, Bioswamp), ACK1 (1:1000, PAB36364-P), p-AKT (Thr308) (1:1000, PAB43323-P, Bioswamp), p-AKT (Ser473) (1:1000, 43181-P, Bioswamp), AKT (1:1000, PAB30596, Bioswamp), pro-caspase 3 (1:10000, ab32499, Abcam, Cambridge, UK), cleaved caspase 3 (1:1000, MAB37300, Bioswamp), and glyceraldehyde 3-phosphate dehydrogenase (GAPDH, 1:1000, PAB36269, Bioswamp).

### Statistical analysis

All experiments were performed in triplicate (n = 3) and the results are presented as the mean ± standard deviation (SD). Comparisons between control and treatment groups were performed using the one-sample t-test using OriginPro 8.0 (OriginLab Corporation, Northampton, MA, USA). A value of *p* < 0.05 indicates statistical significance.

## RESULTS

### Dose-dependent effect of single ACK1 and AKT inhibition on NSCLC cell viability

**FIGURE 1:**
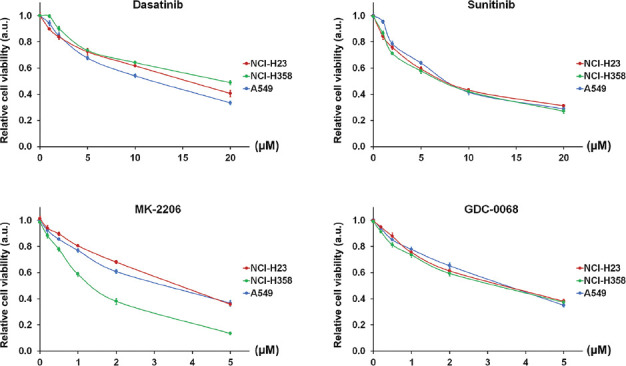
Individual effect of activated CDC42-associated kinase 1 (ACK1) and protein kinase B (AKT) inhibition on non-small-cell lung cancer (NSCLC) cell viability. NSCLC cell lines NCI-H23, NCI-H358, and A549 were treated individually with inhibitors of either ACK1 (dasatinib, sunitinib) or AKT (MK-2206, GDC-0068) at various concentrations (0, 1, 2, 5, 10, and 20 μM for dasat­inib and sunitinib; 0, 0.2, 0.5, 1, 2, and 5 μM for MK-2206 and GDC-0068). Cell viability was evaluated using MTT assay after 48 hours of culture with each drug. Inhibitors of both ACK1 and AKT induced a sharp decrease in the viability of NCI-H23, NCI-H358, and A549 cells in a concentration-dependent manner. The results are shown as the mean ± SD (n = 3, t-test). IC_25_ and IC_50_ represent quarter and half maximal inhibitory concentration, respectively; a.u.: arbitrary units.

The downstream effects of ACK1 and/or AKT inhibition were investigated in three *KRAS*-mutant NSCLC cell lines (NCI-H23, NCI-H358, and A549). The inhibitors of ACK1 (dasatinib and sunitinib) and AKT (MK-2206 and GDC-0068) were selected as model drugs for this purpose. We first evaluated the individual effects of each drug on NSCLC cell ­viability by treating each cell line separately with each drug at various concentrations (0, 1, 2, 5, 10, and 20 μM for dasatinib and sunitinib; 0, 0.2, 0.5, 1, 2, and 5 μM for MK-2206 and GDC-0068). As demonstrated in Figure 1, the inhibitors of both ACK1 and AKT induced a sharp decrease in the viability of NCI-H23, NCI-H358, and A549 cells in a concentration-dependent manner. Accordingly, we calculated the IC_25_ and IC_50_ values of each drug pertaining to each cell line from the cell viability curves (Table 1).

**TABLE 1:**
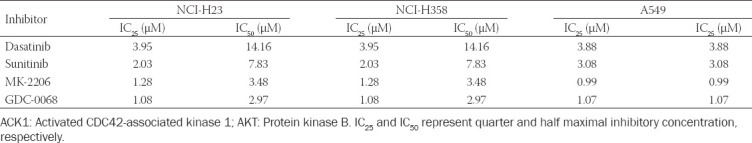
IC_25_ and IC_50_ values of ACK1 and AKT inhibitors in NCI-H23, NCI-H358, and A549 cells

### Selection of optimal drug combination using the Chou-Talalay equation

We next employed the Chou-Talalay method [19] to examine the effect of drug combination and to calculate the optimal combination of ACK1 and AKT inhibition in suppressing NSCLC cell growth. The Chou-Talalay equation yields the combination index (CI) of two drugs and is defined as:

CI = D1/D1_IC50_ + D2/D2_IC50_ + (D1 × D2)/(D1_IC50_ × D2_IC50_) (Eq. 1)

Where D1 and D2 represent the applied concentrations of the two drugs and D1_IC50_ and D2_IC50_ represent the IC_50_ values of the two drugs. For this study, we combined an ACK1 inhibitor with an AKT inhibitor, resulting in four drug combinations (dasatinib + MK2206, dasatinib + GDC-0068, sunitinib + MK-2206, and sunitinib + GDC-0068). Using Eq. 1, we calculated the CI of all drug combinations by substituting D1 and D2 with the respective IC_25_ and IC_50_ values of each individual drug (Table 2). Further, because CI < 1 indicates a synergistic relationship between two drugs [20], we screened for the optimal drug combination by selecting the lowest CI for each cell line. For both NCI-H23 and NCI-H358, the combination of sunitinib (S) at IC_25_ and GDC-0068 (G) at IC_25_ yielded the lowest CI value (0.71 and 0.64, respectively). For A549, the combination of dasatinib (D) at IC_25_ and MK-2206 (M) at IC_25_ yielded a CI of 0.80. These combinations of drug concentrations were applied in the subsequent experiments.

**TABLE 2:**
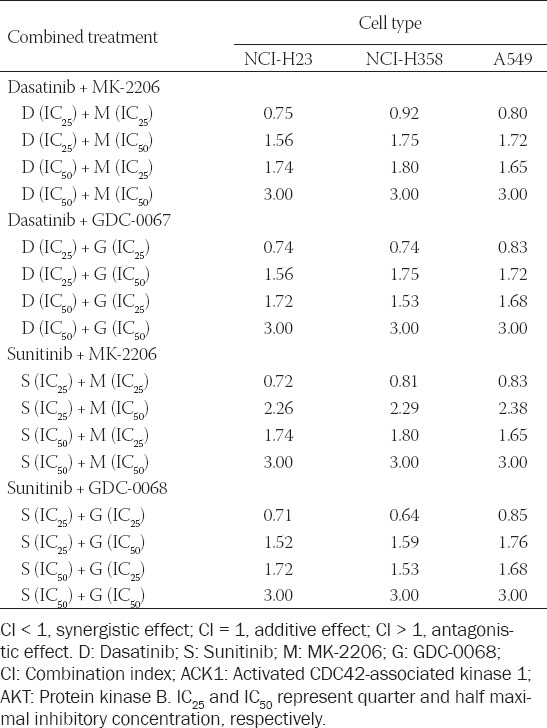
CI of the effect of combined ACK1 and AKT inhibition in NCI-H23, NCI-H358, and A549 cells, calculated using the Chou-Talalay equation

### Effect of combined ACK1 and AKT inhibition on NSCLC cell viability

**FIGURE 2:**
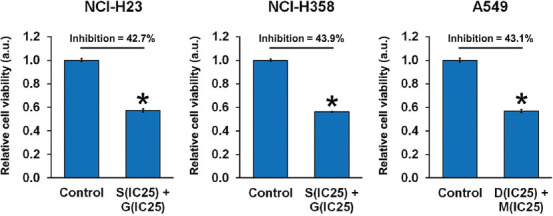
Combined effect of activated CDC42-associated kinase 1 (ACK1) and protein kinase B (AKT) inhibition on non-small-cell lung cancer (NSCLC) cell viability. NSCLC cell lines NCI-H23, NCI-H358, and A549 were treated with combinations of an ACK1 inhibitor (dasatinib, D; sunitinib, S) and an AKT inhibitor (MK-2206, M; GDC-0068, G) at the calculated optimal concentrations. Cell viability was evaluated using MTT assay after 48 hours of culture with each drug combination. For NCI-H23 and NCI-H358 cells, sunitinib at IC_25_ (2.03 μM and 1.98 μM, respectively) combined with GDC-0068 at IC_25_ (1.08 μM and 0.80 μM, respectively) significantly suppressed cell viability by 42.7% and 43.9%, respectively, after 48 h of treatment. For A549 cells, dasatinib at IC_25_ (3.88 μM) combined with MK-2206 at IC_25_ (0.99 μM) significantly suppressed cell viability by 43.1% after 48 h of treatment. The results are shown as the mean ± SD (n = 3, t-test), *p < 0.05 vs. control. IC_25_ represents the quarter maximal inhibitory concentration; a.u.: arbitrary units.

Using the calculated concentrations for each drug combination that exhibit the most optimal synergistic effect, we evaluated the effect of combined ACK1 and AKT inhibition on the viability of NSCLC cells (Figure 2). For NCI-H23 and NCI-H358 cells, sunitinib at IC_25_ (2.03 μM and 1.98 μM, respectively) combined with GDC-0068 at IC_25_ (1.08 μM and 0.80 μM, respectively) significantly suppressed cell viability by 42.7% and 43.9%, respectively, after 48 hours of treatment. For A549 cells, dasatinib at IC_25_ (3.88 μM) combined with MK-2206 at IC_25_ (0.99 μM) significantly suppressed cell viability by 43.1% after 48 hours of treatment.

### Effect of combined ACK1 and AKT inhibition on NSCLC cell apoptosis and cell cycle progression

**FIGURE 3:**
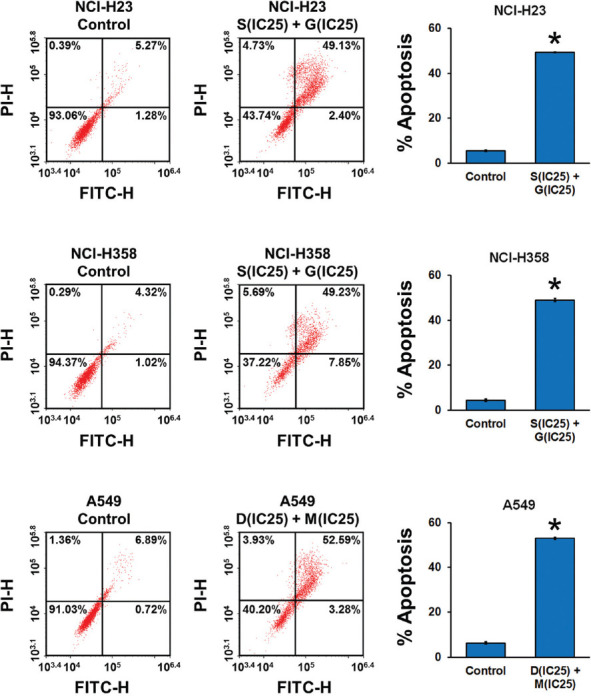
Combined effect of activated CDC42-associated kinase 1 (ACK1) and protein kinase B (AKT) inhibition on non-small-cell lung cancer (NSCLC) cell apoptosis. NSCLC cell lines NCI-H23, NCI-H358, and A549 were treated with combinations of an ACK1 inhibitor (dasatinib, D; sunitinib, S) and an AKT inhibitor (MK-2206, M; GDC-0068, G) at the calculated optimal concen­trations. Cell apoptosis was evaluated using flow cytometry after 24 hours of culture with each drug combination. Bar graphs illustrate the percentage of late apoptotic cells (the upper right quadrant in the flow cytometry plot). The results of flow cytometry revealed that combined treatment with ACK1 and AKT inhibitors at optimal concentrations significantly increased the late-apoptotic population of NCI-H23, NCI-H358, and A549 cells. The results are shown as the mean ± SD (n = 3, t-test), *p < 0.05 vs. control. IC_25_ represents the quarter maximal inhibitory concentration; FITC: fluorescein isothiocyanate; PI: propidium iodide.

**FIGURE 4:**
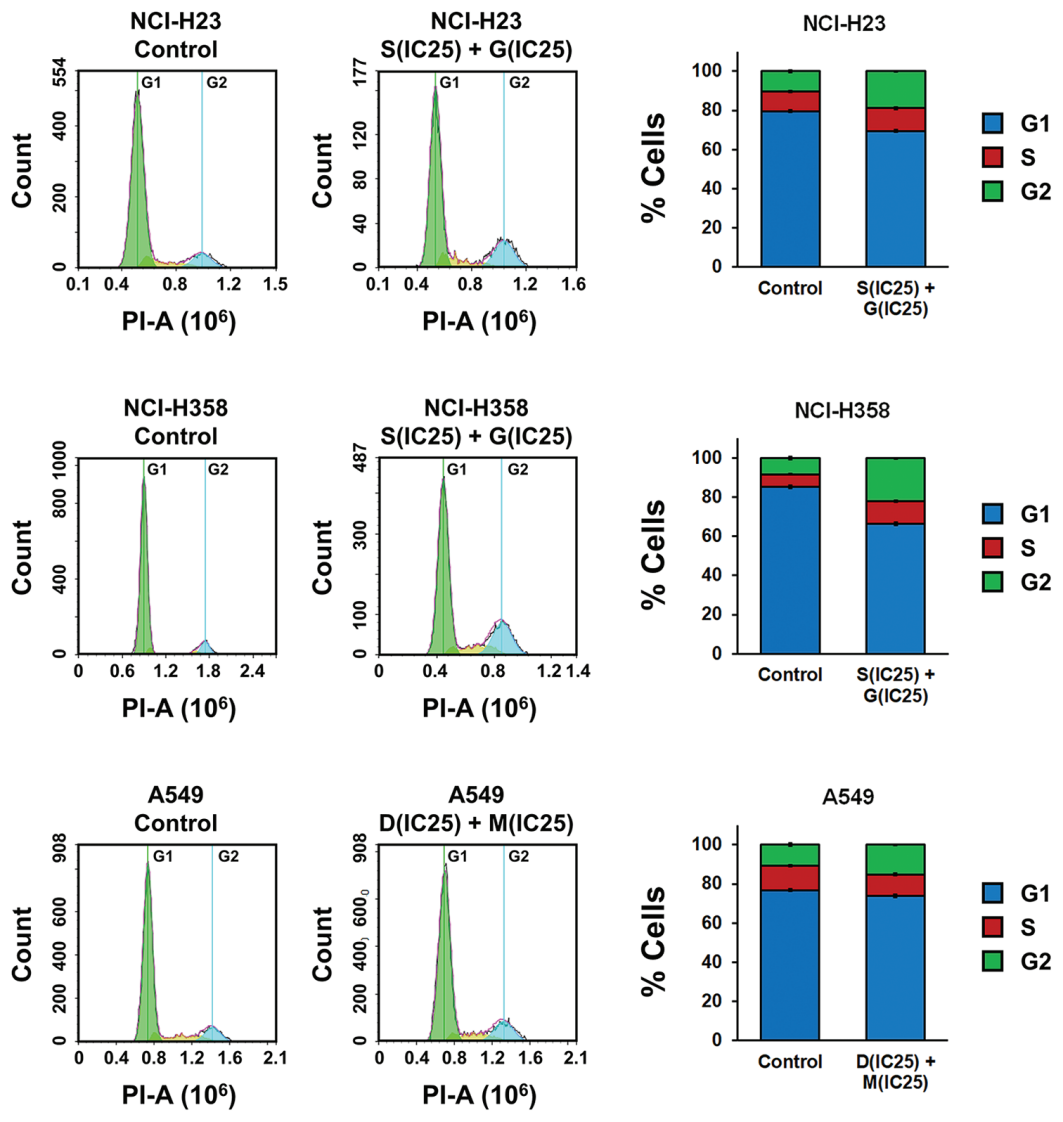
Combined effect of activated CDC42-associated kinase 1 (ACK1) and protein kinase B (AKT) inhibition on cell cycle progression in non-small-cell lung cancer (NSCLC) cells. NSCLC cell lines NCI-H23, NCI-H358, and A549 were treated with combi­nations of an ACK1 inhibitor (dasatinib, D; sunitinib, S) and an AKT inhibitor (MK-2206, M; GDC-0068, G) at the calculated optimal concentrations. Cell cycle progression was evaluated using flow cytometry after 48 hours of culture with each drug combination. With combined ACK1 and AKT inhibitor treatment, the percentage of NSCLC cells in the G1 phase was decreased, whereas that in the G2 phase showed a clear increase. The results are shown as the mean ± SD (n = 3, t-test). IC_25_ represents the quarter maximal inhibitory concentration; PI: propidium iodide.

We then assessed the effect of combined ACK1 and AKT inhibition on NSCLC cell apoptosis and cell cycle progression. The results of flow cytometry revealed that combined treatment with ACK1 and AKT inhibitors at optimal concentrations significantly increased the late-apoptotic population of NCI-H23, NCI-H358, and A549 cells (Figure 3). Meanwhile, a shift was observed in the proportions of cells in each phase of the cell cycle (Figure 4). With combined ACK1 and AKT inhibitor treatment, the percentage of NSCLC cells in the G1 phase was decreased, whereas that in the G2 phase showed a clear increase. These results indicate that combined ACK1 and AKT inhibition promoted apoptosis by inducing cell cycle arrest at the G2 phase.

### Effect of combined ACK1 and AKT inhibition on NSCLC cell migration and invasion

**FIGURE 5:**
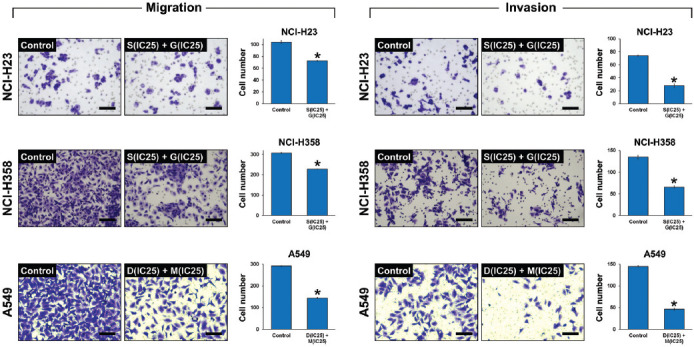
Combined effect of activated CDC42-associated kinase 1 (ACK1) and protein kinase B (AKT) inhibition on non-small-cell lung cancer (NSCLC) cell migration and invasion. NSCLC cell lines NCI-H23, NCI-H358, and A549 were treated with combinations of an ACK1 inhibitor (dasatinib, D; sunitinib, S) and an AKT inhibitor (MK-2206, M; GDC-0068, G) at the calculated optimal concentrations. Cell migration and invasion were evaluated using Transwell assay after 48 hours of culture with each drug combination. The combined treatment with ACK1 and AKT inhibitors at optimal concentrations caused significantly impaired migration of NCI-H23, NCI-H358, and A549 cells by 30.1%, 25.8%, and 50.5%, respectively, compared to that of control cells. Meanwhile, the invasion of these cells was reduced by 62.2%, 51.2%, and 68.0%, respectively. Scale bar = 100 μM. The results are shown as the mean ± SD (n = 3, t-test), *p < 0.05 vs. control. IC_25_ represents the quarter maximal inhibitory concentration.

We next investigated the effect of combined ACK1 and AKT inhibition on the ability of NSCLC cells to migrate and invade (Figure 5). Using Transwell assays, we observed that the combined treatment with ACK1 and AKT inhibitors at optimal concentrations caused significantly impaired migration of NCI-H23, NCI-H358, and A549 cells by 30.1%, 25.8%, and 50.5%, respectively, compared to that of control cells. Meanwhile, invasion of these cells was reduced by 62.2%, 51.2%, and 68.0%, respectively.

### Signaling pathways involved in the effect of ACK1 and AKT inhibition on NSCLC cell behavior

**FIGURE 6:**
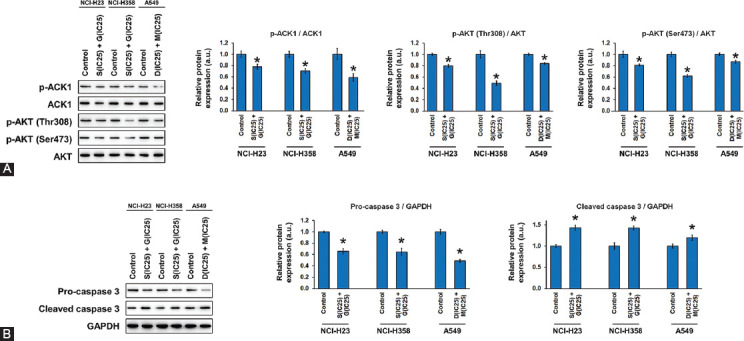
Combined effect of activated CDC42-associated kinase 1 (ACK1) and protein kinase B (AKT) inhibition on protein expression in non-small-cell lung cancer (NSCLC) cells. NSCLC cell lines NCI-H23, NCI-H358, and A549 were treated with combi­nations of an ACK1 inhibitor (dasatinib, D; sunitinib, S) and an AKT inhibitor (MK-2206, M; GDC-0068, G) at the calculated opti­mal concentrations. Protein expression was evaluated using western blot after 24 hours of culture with each drug combination. (A) The phosphorylation levels of ACK1 and AKT (at Thr308 and Ser473) are expressed relative to the total protein level of ACK1 and AKT, respectively. (B) The protein levels of pro-caspase 3 and cleaved caspase 3 are expressed relative to that of GAPDH as an internal control. The combined ACK1 and AKT inhibition exerted a drastic effect on NSCLC cell apoptosis by regulating the ACK1/AKT signaling pathways. The results are shown as the mean ± SD (n = 3, t-test), *p < 0.05 vs. control. IC_25_ represents the quarter maximal inhibitory concentration. GAPDH: Glyceraldehyde 3-phosphate dehydrogenase.

Finally, we examined whether the combined inhibition of ACK1 and AKT yielded an effect on the expression of proteins involved in the ACK1/AKT pathway and apoptotic signaling. For ACK1/AKT signaling, we evaluated the effect of drugs on protein phosphorylation, which is an indication of signaling pathway activation (Figure 6A). In all cases, the combined treatment with the optimal concentrations of ACK1 and AKT inhibitors reduced the level of ACK1 phosphorylation with respect to the total ACK1 protein content. Similarly, the phosphorylation of AKT at the Thr308 and Ser473 sites showed a significant decline with respect to the total AKT protein content. In terms of apoptotic signaling, we looked closely at the relative levels of pro-caspase 3 and cleaved caspase 3 before and after the combined inhibitor treatment (Figure 6B). Consistent with the results of apoptosis obtained by flow cytometry, the expression of pro-caspase 3 was downregulated by drug treatment, whereas that of cleaved caspase 3 was upregulated. These findings implicate that the combined ACK1 and AKT inhibition exerted a drastic effect on NSCLC cell apoptosis by regulating the ACK1/AKT signaling pathways.

## DISCUSSION

Recent strategies in lung cancer treatment have shifted attention to targeted therapy as an alternative to traditional modalities in an attempt to develop selective and effective treatment schemes. The identification of “driver genes” and oncogenic driver mutations is critical for such purpose [21,22]. In the case of NSCLC, the *KRAS* mutation has emerged as a clinical challenge to be addressed, as *KRAS*-mutant lung cancer often causes complications in systemic anti-cancer drug therapy [23]. Therefore, the search for an effective method of overcoming drug resistance associated with the *KRAS* mutation has become an important area of research.

Activated *ACK1* has been identified as an oncogene in a variety of cancers, including prostate, ovarian, and lung cancer [18]. Its activity is closely linked to that of AKT, the activation of which is widely implicated in many malignancies. ACK1-induced activation of AKT is a prominent factor not only in lung cancer but also in gastric cancer [24], breast cancer [11], and hepatocellular carcinoma [25]. Because of the promising therapeutic potential of AKT inhibition in cancer treatment, drugs that act as inhibitors of AKT, such as MK-2206 and GDC-0068 used in this study, have undergone clinical trial [26,27]. The successful inhibition of AKT is evaluated by assessing the phosphorylation of AKT at Ser473 or Thr308 [18]. In the same manner, inhibition of ACK1 is expected to exert a therapeutic effect against lung cancer, but the ideal ACK1 inhibitor remains elusive. It is worth nothing that dasatinib and sunitinib are not specific inhibitors of ACK1. While dasatinib blocks ACK1, it also inhibits Src family kinases and EphA2 receptor tyrosine kinase to suppress human melanoma cell migration and invasion [28]. Sunitinib inhibits a variety of other receptor tyrosine kinases, including vascular endothelial growth factor receptors 1-3 and PDGF-R, and has been approved for the treatment of imatinib-resistant tumors [29].

In this study, we treated three NSCLC cell lines with dasatinib or sunitinib [18,30,31] in combination with MK-2206 or GDC-0068. The Chou-Talalay method was employed to assess the difference in the effect of drug combination and to determine the optimal concentrations of ACK1 and AKT inhibitor for NSCLC treatment. Using the Chou-Talalay method to calculate the CI of two drugs, we were able to identify whether this drug combination at specific concentrations exhibits additive (CI = 1), synergistic (CI < 1), or antagonistic (CI > 1) effects [20]. Having screened for the optimal combination of ACK1/AKT inhibitors that yielded the lowest CI in the three *KRAS*-mutant NSCLC cell lines tested (NCI-H23, NCI-H358, and A549), we proceeded to evaluate the effect of the drug combinations on various cell behaviors. We observed that the combined ACK1/AKT inhibition significantly suppressed the viability, migration, and invasion of NSCLC cells while promoting apoptosis. As expected, these phenomena were associated with the simultaneous decline in the phosphorylation levels of ACK1 and AKT (at both Ser473 and Thr308), as well as in the enhanced expression of cleaved caspase 3.

Our findings are supported by those of Tan et al., who found that inhibition of ACK1 using bosutinib had an inhibitory effect on the migration and invasion of NSCLC cell lines [17]. Interestingly, the inhibitory effect seemed to be only present in *KRAS*-mutant cells and not *KRAS*-wild type cells, suggesting the specificity of ACK1 inhibition against the *KRAS* mutation. However, the results of our study seem to contradict those of Rao et al., who reported that inhibition of AKT1 signaling using MK-2206 promoted *KRAS*-mutant lung cancer cell invasion and metastasis [32]. This may be due to the conflicting role of AKT in various cancers under different circumstances, which have been vastly reported in the literature. AKT is generally known to be highly expressed in cancer and reportedly mediates important processes such as cell survival and cell cycle progression [33,34]. However, in some cases, overexpression of AKT may surprisingly lead to decreased tumor invasion and motility [35,36]. The applied dose of the AKT inhibitor MK-2206 may also play a role in determining cell behavior. As suggested by Rao et al., low doses of MK-2206 exerted a promoting effect on NSCLC cell invasiveness, whereas high doses significantly reduced cell viability [32]. In our study, the combined inhibition of ACK1 and AKT exhibited a potent synergistic effect against NSCLC survival, migration, and invasion. Given that AKT is activated by ACK1, it is reasonable to propose that ACK1 inhibition acts as an enhanced mechanism of suppressing the oncogenic role of AKT, along with MK-2206 as an inhibitor of AKT.

Taken together, our results support the therapeutic potential of combined ACK1/AKT inhibition as a strategy against *KRAS*-mutant NSCLC. Our findings provide the basis for the clinical translation of promising biological targeted drugs (ACK1 and AKT inhibitors) and their rational combination in cancer treatment.

## References

[1] Siegel RL, Miller KD, Jemal A (2020). Cancer statistics, 2020. CA Cancer J Clin.

[2] Zappa C, Mousa SA (2016). Non-small cell lung cancer:Current treatment and future advances. Transl Lung Cancer Res.

[3] Ferrer I, Zugazagoitia J, Herbertz S, John W, Paz-Ares L, Schmid-Bindert G (2018). KRAS-mutant non-small cell lung cancer: From biology to therapy. Lung Cancer.

[4] Lee T, Lee B, Choi YL, Han J, Ahn MJ, Um SW (2016). Non-small cell lung cancer with concomitant EGFR, KRAS, and ALK Mutation: Clinicopathologic features of 12 cases. J Pathol Transl Med.

[5] Bhattacharya S, Socinski MA, Burns TF (2015). KRAS mutant lung cancer:Progress thus far on an elusive therapeutic target. Clin Transl Med.

[6] Xu K, Park D, Magis AT, Zhang J, Zhou W, Sica GL (2019). Small molecule KRAS agonist for mutant KRAS cancer therapy. Mol Cancer.

[7] Pao W, Wang TY, Riely GJ, Miller VA, Pan Q, Ladanyi M (2005). KRAS mutations and primary resistance of lung adenocarcinomas to gefitinib or erlotinib. PLoS Med.

[8] Pikor LA, Ramnarine VR, Lam S, Lam WL (2013). Genetic alterations defining NSCLC subtypes and their therapeutic implications. Lung Cancer.

[9] Stahel R, Peters S, Baas P, Brambilla E, Cappuzzo F, De Ruysscher D (2013). Strategies for improving outcomes in NSCLC:A look to the future. Lung Cancer.

[10] Mahajan K, Mahajan NP (2010). Shepherding AKT and androgen receptor by Ack1 tyrosine kinase. J Cell Physiol.

[11] Mahajan K, Coppola D, Challa S, Fang B, Chen YA, Zhu W (2010). Ack1 mediated AKT/PKB tyrosine 176 phosphorylation regulates its activation. PLoS One.

[12] Xu SH, Huang JZ, Chen M, Zeng M, Zou FY, Chen D (2017). Amplification of ACK1 promotes gastric tumorigenesis via ECD-dependent p53 ubiquitination degradation. Oncotarget.

[13] Mahajan K, Malla P, Lawrence HR, Chen Z, Kumar-Sinha C, Malik R (2017). ACK1/TNK2 regulates histone H4 Tyr88-phosphorylation and AR gene expression in castration-resistant prostate cancer. Cancer Cell.

[14] Hu F, Liu H, Xie X, Mei J, Wang M (2016). Activated CDC42-associated kinase is up-regulated in non-small-cell lung cancer and necessary for FGFR-mediated AKT activation. Mol Carcinog.

[15] Mahajan NP, Whang YE, Mohler JL, Earp HS (2005). Activated tyrosine kinase Ack1 promotes prostate tumorigenesis:Role of Ack1 in polyubiquitination of tumor suppressor Wwox. Cancer Res.

[16] Chan W, Tian R, Lee YF, Sit ST, Lim L, Manser E (2009). Down-regulation of active ACK1 is mediated by association with the E3 ubiquitin ligase Nedd4-2. J Biol Chem.

[17] Tan DS, Haaland B, Gan JM, Tham SC, Sinha I, Tan EH (2014). Bosutinib inhibits migration and invasion via ACK1 in KRAS mutant non-small cell lung cancer. Mol Cancer.

[18] Mahajan K, Mahajan NP (2013). ACK1 tyrosine kinase:Targeted inhibition to block cancer cell proliferation. Cancer Lett.

[19] Chou TC, Talalay P (1984). Quantitative analysis of dose-effect relationships:The combined effects of multiple drugs or enzyme inhibitors. Adv Enzyme Regul.

[20] Chou TC (2010). Drug combination studies and their synergy quantification using the Chou-Talalay method. Cancer Res.

[21] Guo Y, Cao R, Zhang X, Huang L, Sun L, Zhao J (2019). Recent progress in rare oncogenic drivers and targeted therapy for non-small cell lung cancer. Onco Targets Ther.

[22] Luo SY, Lam DC (2013). Oncogenic driver mutations in lung cancer. Transl Respir Med.

[23] Zarredar H, Pashapour S, Ansarin K, Khalili M, Baghban R, Farajnia S (2019). Combination therapy with KRAS siRNA and EGFR inhibitor AZD8931 suppresses lung cancer cell growth *in vitro*. J Cell Physiol.

[24] Xu SH, Huang JZ, Xu ML, Yu G, Yin XF, Chen D (2015). ACK1 promotes gastric cancer epithelial-mesenchymal transition and metastasis through AKT-POU2F1-ECD signalling. J Pathol.

[25] Xie B, Zen Q, Wang X, He X, Xie Y, Zhang Z (2015). ACK1 promotes hepatocellular carcinoma progression via downregulating WWOX and activating AKT signaling. Int J Oncol.

[26] Pal SK, Reckamp K, Yu H, Figlin RA (2010). Akt inhibitors in clinical development for the treatment of cancer. Expert Opin Investig Drugs.

[27] de Bono JS, De Giorgi U, Rodrigues DN, Massard C, Bracarda S, Font A (2019). Randomized phase II study evaluating AKT blockade with ipatasertib, in combination with abiraterone, in patients with metastatic prostate cancer with and without PTEN loss. Clin Cancer Res.

[28] Buettner R, Mesa T, Vultur A, Lee F, Jove R (2008). Inhibition of Src family kinases with dasatinib blocks migration and invasion of human melanoma cells. Mol Cancer Res.

[29] Roskoski R (2007). Sunitinib:A VEGF and PDGF receptor protein kinase and angiogenesis inhibitor. Biochem Biophys Res Commun.

[30] Liu Y, Karaca M, Zhang Z, Gioeli D, Earp HS, Whang YE (2010). Dasatinib inhibits site-specific tyrosine phosphorylation of androgen receptor by Ack1 and Src kinases. Oncogene.

[31] Phatak SS, Zhang S (2013). A novel multi-modal drug repurposing approach for identification of potent ACK1 inhibitors. Pac Symp Biocomput.

[32] Rao G, Pierobon M, Kim IK, Hsu WH, Deng J, Moon YW (2017). Inhibition of AKT1 signaling promotes invasion and metastasis of non-small cell lung cancer cells with K-RAS or EGFR mutations. Sci Rep.

[33] Altomare DA, Testa JR (2005). Perturbations of the AKT signaling pathway in human cancer. Oncogene.

[34] Mundi PS, Sachdev J, McCourt C, Kalinsky K (2016). AKT in cancer:New molecular insights and advances in drug development. Br J Clin Pharmacol.

[35] Yoeli-Lerner M, Yiu GK, Rabinovitz I, Erhardt P, Jauliac S, Toker A (2005). Akt blocks breast cancer cell motility and invasion through the transcription factor NFAT. Mol Cell.

[36] Liu H, Radisky DC, Nelson CM, Zhang H, Fata JE, Roth RA (2006). Mechanism of Akt1 inhibition of breast cancer cell invasion reveals a protumorigenic role for TSC2. Proc Natl Acad Sci U S A.

